# Safety Compliance in a Sample of Italian Mechanical Companies: The Role of Knowledge and Safety Climate

**DOI:** 10.3390/ejihpe12030020

**Published:** 2022-03-04

**Authors:** Federico Ricci, Chiara Panari, Annalisa Pelosi

**Affiliations:** 1 Department of Biomedical, Metabolical and Neurosciences, University of Modena and Reggio Emilia, Via Giuseppe Campi, 287, 41125 Modena, Italy; 2 Department of Economics and Management, University of Parma, Via John Fitzgerald Kennedy, 6, 43125 Parma, Italy; chiara.panari@unipr.it; 3 Department of Medicine and Surgery, University of Parma, Via Gramsci, 14, 43126 Parma, Italy; annalisa.pelosi@unipr.it

**Keywords:** safety training, knowledge, attitudes, behaviours, safety compliance, safety climate

## Abstract

The accident rate in the Italian mechanical sector is still too high, and evidence-based interventions to improve safety performance are essential. To better address this, our study contributes to the understanding of how to promote safety compliance through safe behaviours by using a sample of Italian mechanical workers (n = 109). Before and after scheduled safety training, intervention data on organizational factors, as well as on individual factors affecting safety-related behaviours, were collected. Particularly, data were collected using multiple sources, including self-perception questionnaires (to measure the safety climate among the management and colleagues and the safety attitude), paper and pencil tests (to measure safety knowledge), and observations by personnel with experience in observation tasks (to measure safety behaviours objectively). A model class of competing general linear models was built to determine which of the models was best suited for predicting safety-related behaviours. The results showed that both knowledge and the management’s safety climate effectively promoted safety compliance. Crucial implications for the effectiveness of active teaching methods, along with the need for continuous training and the prominent role of the management team members in giving, through their actions, further relevance to the need to respect rules and procedures, were revealed. Finally, practical implications for researchers, corporate decision makers, government agencies, and international bodies are discussed.

## 1. Introduction

In 2018, across all 27 current members of the EU, there were 2.21 fatal accidents (2.7 in Italy) [1] and 1769 non-fatal accidents (Italy’s index is below the EU average) per 100,000 employed persons [2]. Despite industrial activities accounting for many of the largest decreases in incidence rates of fatal and non-fatal accidents [3] between 2010 and 2018, there are still too many events that cause serious or irreparable damage to workers.

The organizational literature has pointed out the fact that only multi-causal models can provide an exhaustive explanation for adverse events as a symptom of a malfunctioning socio-technical system, which is seen as the interaction of human beings with the system and the social environment. Along this line, recent reviews of the safety literature have emphasized the influence of organizational factors on accidents and near-misses, pointing at the need to shift the interest from individual behaviours to the contexts in which accidents and near-misses occur [4]. Though it is not possible to completely eliminate human error, it is nonetheless necessary to focus our attention on the conditions that may lead to adverse events. We must, therefore, consider the context, task, and characteristics of the operator that may contribute to unsafe behaviours and reduce safety performance [5].

Safe behaviours, as conceptualized by Neil and Griffin [6] and recently confirmed by Kalteh et al. [7], Seo et al. [8], and Kapp [9], can be defined as work performance characterized by two dimensions: safety compliance (with rules and procedures, related to the task) and safety participation (aimed at proactively promoting safety as a value and not as a fulfilment of obligations, related to the context). Additionally, Christian et al. [10] considered safety performance as a synonym of safe behaviour, which is produced by safety knowledge and safety motivation, and they concluded that safety performance affected both accidents and injuries.

Moreover, other research [11] considered safety performance as an observed worker’s engagement in a given behaviour when that behaviour is appropriate for the situation. In a study that explored the relationship between safety culture and safety performance in U.S. nuclear power operations [12], it was noted that safety performance included behavioural and non-behavioural measures alike. In this sense, an Italian study [13] featuring a sample of police officers showed that, through targeted safety training, the officers learned about the basic requirements of sleep and its importance for health and safety. They also learned how to identify symptoms of sleep disorders, how to identify and cope with sleepiness, and how to improve sleep hygiene on a day-to-day basis. All of the above resulted in improved safety compliance, leading to significant improvements in sleep quantity and quality. The authors also reported that there was a marked decrease in the frequency of occupational accidents and near-misses, although they could not demonstrate that there was a causal relationship between safety compliance and objective events. The possible absence of such events is not synonymous with safety; in fact, the measure of safety is not the absence of health damage, but rather the presence of safe behaviour. It is central to consider safe behaviours as indicators of safety, as these help to prevent real damage and to understand whether there is any absence or presence of safety [4]. From a Safety-II perspective [14], while accidents clearly indicate the absence of safety, a lack of accidents cannot necessarily be used to infer a low probability of harm. In this case, safety performance is not dependent on ensuring that “*as few things as possible go wrong*”, but ensuring that “*as many things as possible go right*” ([14], p. 5). The Safety-II perspective assumes that things go well because human behaviours, as a necessary resource for safety performance, can provide the necessary adaptations to adequately respond to changing conditions. In addition to the reasons just mentioned, there are also methodological implications suggesting that the incidence of adverse events should not be used as an indicator of good safety performance. In fact, within the body of evidence of an important systematic review [15], only three studies demonstrated the effectiveness of safety training when referring to such indicators: a clinical evaluation of dermatitis for workers in geriatric facilities; an estimate based on 6 months of injury reporting by farmers; the effect, after one year, on injuries caused by the use of cutters as reported by workers in grocery stores. This is not surprising, given that a previous meta-analysis [16] found that the number of studies examining occupational diseases, injuries, and accidents was insufficient to allow for separate considerations. As indicators, the aforesaid categories of adverse events were less likely to be affected by training, as there were various intervening and time-related variables that could impact the affected adverse events. In line with what was mentioned above, a more recent meta-analytic study [17] confirmed that, in the 28 studies found to be eligible, the use of data on the incidence of adverse events was the least common choice for evaluating the effectiveness of safety training, accounting for 4.5% of the collected measures, and was slightly surpassed (6.8%) by physiological data on body functioning. There is also a limit to the sensitivity of documentary data, which do not tend to reveal significant post-training changes in contexts where accident events are, historically speaking, rare because of the number of workers considered and/or because of the working conditions. An example when such a sensitivity limit was very attenuated was found in an Italian study [18] that involved a population of 2795 workers employed on the construction sites of the Turin–Novara high-speed railway line between July 2002 and July 2005. Even in this case, the authors concluded that the training intervention had a moderate effect on reducing the number of injuries.

### The Antecedents of Safety Compliance

In our manuscript, we focused on the precursors of safety compliance, which were seen as workers’ behaviours related to their tasks, and we analysed the data associated with the impact of safety training on safety knowledge, attitude, and work climate. Studies on the effectiveness of safety training, as reflected in original research and in published reviews and meta-analyses [15,16,17], are now clearly focused on assessing the changes concerning knowledge and attitudes after safety training has been conducted. Furthermore, we believe that the effect of training on the safety climate at work must be taken into consideration because this construct, which refers to the shared perceptions of safety’s value in an organization, also depends on the organization’s investment in safety training and updating [19,20,21].

The pivotal contribution of Griffin and Neal (2000) [6] lies in the suggestion that safety knowledge is a precursor of safety compliance, but not an antecedent of safety participation. Studies in this research area contributed to extending the understanding of precursors of safety performance. A recent systematic review [7] concluded that increasing the level of safety climate and safety culture could be effective in reducing incidents and improving safety performance indicators. However, the review added nothing to the notion of knowledge as a precursor of safety performance. A previous meta-analytical contribution [10] of 90 studies on safety performance in working contexts supported a full-mediation model that identified safety knowledge and safety motivation as precursors of safety performance and as a unique construct overall without considering the specific relationship between safety compliance and safety participation. To understand the individual and organizational factors that influenced compliance, Hu et al. [22] enriched safety performance models by considering cognitive–motivational factors that influenced safety compliance. However, they did not evaluate the contribution of safety knowledge to safety compliance. On the other hand, Smith-Crowe et al. [23] disconfirmed that organizational climate was as a moderator in safety knowledge–safety performance relationships. In conclusion, the body of evidence on safety performance is not complete and the results are not entirely clear.

Consequently, it is necessary to thoroughly examine the relationships that exist among safety knowledge, attitudes, climate, and behaviours. Research showing that the level of safety knowledge was significantly related to safety compliance demonstrated that there was a positive relationship between safety training and activities carried out by individuals to maintain workplace safety and adhere to organizational safety procedures [24].

In fact, safety training played a significant role in the enhancement of different outcomes [15,16,17], thereby increasing safety awareness, reducing the overconfidence bias that led to mistakes [25], and changing the behaviour of employees [26]. Motivational dynamics were also related to attitudes, which were defined as stable personal evaluations—whether favourable or unfavourable—that determined the subjective representation of one’s surroundings [27]. Research has shown that the influence of attitudes on safe behaviours [19] depends on the possibility of the former coming from experience because, in this case, attitudes become more accessible and translate into a higher level of mastery with respect to another person’s observation or verbal communication. A study conducted in an automobile manufacturing plant [28] indicated that workers’ attitudes were precursors of unsafe behaviours related to the conflict between productivity and safety, and that perceptions of being pressured to ensure efficiency predicted occupational accidents. The author then concluded that improvement in safety practice must have affected workers’ attitudes and perceptions of occupational conditions, thus leading to safer behaviours.

Among organizational distal antecedents, data have indicated that a better understanding of safety issues in a specific environment depends on the safety climate. Guldenmund [20] concluded that the safety climate could be considered as an alternative safety performance indicator. This construct of the organizational safety climate refers to: the perceptions that members of the same organization share, such as: the importance attributed to safe behaviour [29]; the measures with which violations of safety regulations are sanctioned; superiors’ attention to compliance with the rules; the organization’s investment in safety training and updating [21]. Jiang et al. [30] demonstrated that providing supervision between colleagues during everyday occupational activities, supported by regular peer exchanges on safety practices (or other interventions to increase the perceived value of safe actions), could increase the frequency of safe behaviour. There is evidence suggesting that a safety climate fosters and increases workers’ motivation to behave safely, that it influences their commitment to safety, and that it is a meaningful predictor of safe behaviours. Reason [31] showed that violations (dangerous actions that expressed an intentional rejection of procedures and rules by a worker in the use of PPE), unlike dangerous actions resulting from cognitive failures, were closely related to the safety climate. That is, the failure to comply with the rules was affected by the workers’ perception of the degree of commitment to health protection that the company showed. The perception that the management was careful with safety, that the organization was concerned with various types of risks, and that safety was a guiding criterion in the management of human resources translated into greater effort and attention to safety issues among workers. The safety climate defined the real regulatory horizon, the rule to which people and groups adapted—that is, the subjective norm [32], the result of the pressure exerted by others (superiors and colleagues) within the workers’ own social group of reference. These subjective rules helped to form individuals’ perceptions of what behaviours (e.g., respect or violation of procedures) were expected and whether they were rewarded in the context that workers belonged to. This scenario is in line with the action regulation theory, according to which people approximate their view of reality by interacting on and with reality [33,34], and that action is influenced by personal, stable, and dynamic factors, as well as by signals and feedback from the physical and social environment [35]. This means that, although a correct perception of risk is necessary, it may not be enough, given that daily behaviour is influenced by informal rules and social conditioning.

Therefore, we considered the safety climate as the company’s response to the need for occupational accident prevention. As clearly indicated by the psychosocial causal model of work-related accidents [36], the safety climate directly determines the practices implemented by supervisors, which, in turn, influence the team’s internal actions, with final repercussions on accident results. Safety climate factors show better values in companies with low injury incidence compared to those with higher rates [37].

Indeed, there is evidence that an improvement in the safety climate actually causes a reduction in the severity of accidents, which is measured in terms of lost working days [38]. Safety climate dimensions affect vessel accidents with respect to crew fatalities and vessel failures in the container shipping context [39], and there is a greater likelihood of accidents in worse safety climates [40]. Furthermore, hospital safety climate is related to indicators of potential safety events [41].

The above-mentioned literature was the basis for our research work, which aimed to analyse the precursors of safety compliance whilst focusing on organizational factors (the management’s safety climate, as well as colleagues’ safety climate) and individual factors (safety knowledge and attitude). In particular, we tested different models to examine the impact of the above four precursors on safety compliance, both individually (H1) and in combination (H2) or in interaction with each other (H3), so as to identify the most predictive factors of safe behaviour. 

It is evident in a large body of studies that there are several accredited models, which are more or less complex, on the antecedents of safety compliance. However, few of these studies presented data showing the differences produced by training on the precursors considered. Moreover, to date, this study is only the second research involving an Italian sample of metalworkers. Unlike the previous one by Brondino [42], our research is the first to examine knowledge as a precursor of safe behaviour. 

## 2. Materials and Methods

### 2.1. Participants and Training Intervention

A total of 109 subjects, working for two companies that voluntarily joined the research (“B”: N = 60; “S”: N = 49), participated in the study, 75 of whom were male (68.8%; B = 66.7%, S = 71.4). Their average age was 45.4 ± 8.7 years (from 23 to 64), and their average seniority was 17.3 ± 8.1 years (0 to 40). No significant differences were found between the companies as regards age (B: 47.5 ± 7.8, S: 43.2 ± 9.2; t107 = 1.278, *p* = 0.102) or seniority (B: 17.5 ± 8.5, S: 17.0 ± 7.5; t107 = 0.344, *p* = 0.731). The subjects’ average schooling was 9.9 ± 1.9 years (B: 11.1 ± 1.4, S: 8.4 ± 1.6). Of the participants, 19 (17.4%) held a role in the Health and Safety Department, with a mild prevalence of S (22.4% versus 13.3%). 

The mechanical companies that joined the study were firms of excellence in the industrial district of the province of Reggio Emilia. These two large companies produced gardening machines (B) and household appliances (S). The management of the companies identified the departments that would be involved in the research by selecting those connected with the assembly of mechanical components. There were no professional relationships between the two sets of workers from the manufacturing plant, as each plant was run by a different company management and the sites were geographically far from each other.

The inclusion criteria included the obligation attending mandatory safety retraining, complying with the Italian law, and having sufficient comprehension of the Italian language. All of the workers employed in the selected departments participated in our ecological study. Therefore, the sampling of the companies was based on the interest shown by the companies themselves in participating in the proposed training course (convenience sampling), but, within the selected companies, all workers had completed the training course, as required by D.Lgs.—Legislative Decree 81/08. Accordingly, all workers took part in every phase of training and data collection, with a return rate of participation of 100%.

The training, which was provided through an integrated method [43], lasted 6 h, consisting of two 3 h sessions over several weeks. Six editions of the course were held, with about 15–25 participants per edition. The contents delivered were based on the adverse events that had been documented or were known to be potentially verifiable at work. The intervention was based on a participatory approach so as to improve the different expected outcomes, as participatory training optimizes cost effectiveness [44]. 

In session 1, the trainer, an expert in andragogy, used tailor-made audio–visual material in order to facilitate discussion between the participants during scheduled breaks in the film. The audio–visual material was made in the workplace of the departments involved so as to encourage the workers to identify either dangerous or safe behaviours, and the material included a good level of educational entertainment [45], as well as subtitles to facilitate the attribution of meaning and the effective use of the information presented [46].

Session 2 aimed at creating opportunities for participants to compare experiences, starting with personal case studies, to achieve the following goals: conceptualizing the factors associated with adverse events, enabling the analysis of the causes, defining the take-home message, and sharing the actions needed to improve safety performance.

### 2.2. Instruments

The measures described below were identified on the basis of the research conducted in this area of investigation [15,16,17].

- Knowledge: An adapted version of the scale proposed by Ricci et al. [47] composed of 20 items characterized by different formats: two items concerning the recognition of safe behaviours presented as a photographic stimulus (e.g., safety signage), with three alternatives and only one correct answer; 15 items requiring the production—with paper and pencil—of the correct answers to questions on the role of prevention and the safety procedures to apply (e.g., “What is the safest behaviour to use in the event of an earthquake tremor?”); three items requiring the recognition of the correct answer, among four alternatives, on the obligations and sanctions that the law imposes on workers (e.g., “Breaking the obligation to correctly use the working equipment and the means of transport, as well as the safety equipment, will be punished with:”).
- Attitudes towards safe practices: A self-evaluation questionnaire consisting of three items (e.g., “People get hurt because they don’t apply the procedures”), adapted from Ricci et al. [47], with responses given on a 10-point Likert-type scale (from 1 “completely disagree” to 10 “completely agree”). Reliability value result (Cronbach alpha coefficient): 0.64 [43].
- Safety climate: The Italian version, in reduced form, of the NOSACQ-50 [48], with seven verbal items rated on a 7-point Likert scale (from 1 “completely disagree” to 7 “completely agree”). The items measured the workers’ perception of the actions taken by the management (e.g., “the management involves the workers in decisions concerning safety”) and by their colleagues (e.g., “the workers of this company help one another to work safely”). Reliability value result (Cronbach alpha coefficient): 0.83 [43].
- Behaviours: An original observation checklist containing four parameters (personal protective equipment, manual handling of loads, posture at work, and pace of work). For each parameter, four indicators were identified: 1 = very good; 2 = quite good; 3 = relatively wrong; and 4 = completely wrong. The survey was completed by personnel with experience in observation tasks, who were trained by the lead researcher before proceeding with the scheduled activities.

### 2.3. Procedure

The data collection took place during the collective sessions of safety training, immediately before the beginning of the training (T_1_), and about three months later (T_2_), right after the end of the course. To reduce distractions, at the beginning of each session, the participants put their electronic devices away in a dedicated place.

The representatives of workers’ safety from each company were actively involved in the research project. Each phase of the research (training and data collection) took place during working hours. The questionnaires were filled in using a rigorously anonymous form. However, in order to pair the participants’ responses over time, each participant was asked to calculate a univocal code according to the criteria established by the researcher. The data collection relating to the behaviour (checklist) was the only one that was not self-administered. This observation and recording of behaviours, which lasted many hours, took place during the week prior to the T_1_ meeting and about three months later, in the week following the T_2_ encounter.

All of the participants freely agreed to take part in the study, signed an informed consent form, and provided their sociodemographic data. All of the research activities were performed in total compliance with the ethical and deontological code of psychologists and with the Declaration of Helsinki.

### 2.4. Data Analysis

Given the substantial homogeneity of the workers’ characteristics, none of the analyses considered the company variable.

We considered the difference (delta) between T_2_ and T_1_ for knowledge, positive attitudes, and safety climate as predictors and the T_2_–T_1_ delta for safety-related behaviours as a dependent variable; thus, a positive delta indicates an increase, and is greater the further it is from zero.

A model class of competing general linear models was built in order to determine the best model with respect to the prediction of safety-related behaviours, i.e., the use of PPE in compliance with safety procedures. In addition to the null model, which included only the constant term and the error term, simple regression models, additive models, and models with modulation effects were considered; the predictors were the deltas of knowledge, attitudes, and safety climate perception. Akaike’s information criterion-corrected (finite-sample AIC-corrected; [49]) was used as an indicator of incremental fit to estimate the residual variance of the model corrected for the number of predictors. In addition to the best model, the confidence set of equally plausible models based on Akaike’s weight (relative likelihood) was also considered for discussion; models with an Akaike’s weight approximately equal to 10% of the weight of the best model were included in the confidence set. 

## 3. Results

**Figure 1:**
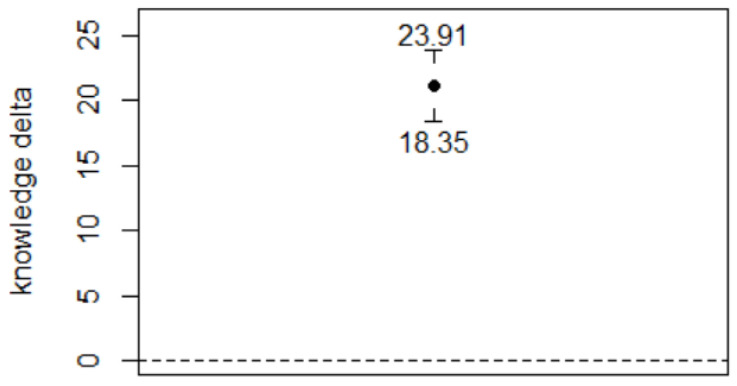
Change in safety-related knowledge (error bars: 95% CI)**.**

Knowledge about safety-related regulations and requirements increased from T_1_ (9.9 ± 3.5 to T_2_ (31.1 ± 16.2), despite a strong variability at T_2_ (∆ = 21.12 ± 14.64, 95% CI 18.34–23.91; Figure 1). 

**Figure 2:**
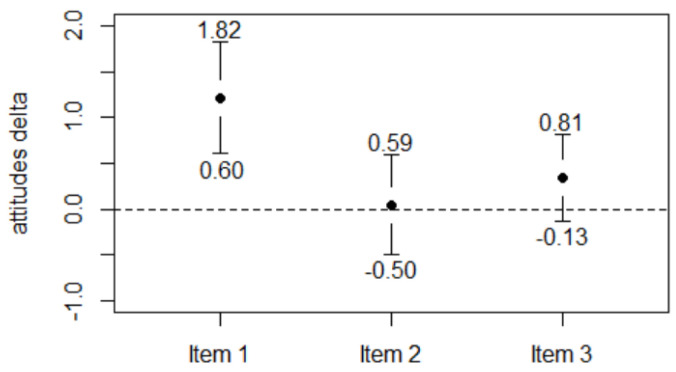
Changes in attitudes towards safety (error bars: 95% CI).

The attitude items (Figure 2) were inconsistent: Item 3 remained unchanged (∆ = 0.34 ± 2.48, 95% CI −0.13–0.82), as did item 2 (∆ = 0.046 ± 2.86, 95% CI −0.49–0.59), whereas item 1 increased (∆ = 1.21 ± 3.2, 95% CI 0.61–1.82), The intra-group variability was substantial, mainly in item 2.

**Figure 3:**
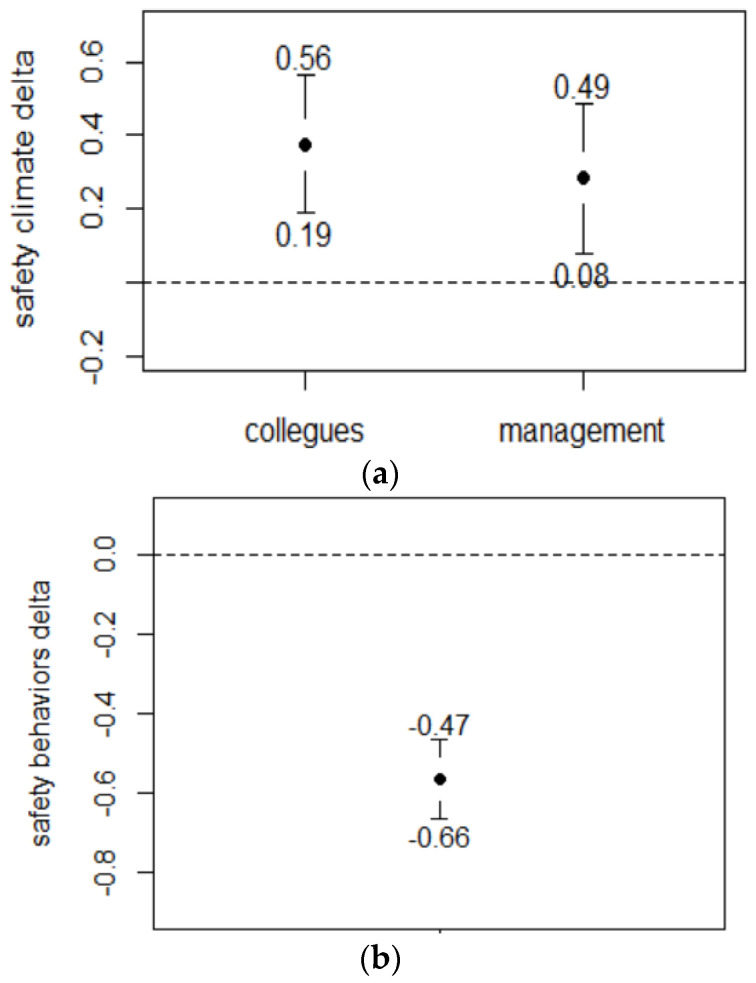
(**a**,**b**) Changes in safety climate and safety-related behaviours.

The change in the perception of the safety climate (Figure 3a) was mild but significant for the dimensions related both to the actions of colleagues (∆ = 0.38 ± 0.98, 95% CI 0.19–0.56) and to the actions of management (∆ = 0.28 ± 1.1, 95% CI 0.08–0.49). The variations in behaviours (Figure 3b) were small, but significant ((∆ = −0.57 ± 0.52, 95% CI −0.66–−0.47).

We then proceeded to create and estimate the hypothesized relationship models. Table 1 shows the incremental fit indices for every model.

The best models were (according to AICc) the knowledge delta plus the safety climate–management actions delta (Model 12), and, even with its clearly lower likelihood, the knowledge delta alone (Model 1); Model 12 had a 52.4% chance of being the best (Akaike’s weight = 0.524) compared to 17.8% for Model 1 (weight = 0.178). Only the former should, therefore, be considered. However, by analogy to the previous model class, we explored the parameters of both models.

In Model 12, the variations in knowledge and safety climate–management explained 28.3% of the variation in the procedures (R^2^ = 0.283, R^2^_adj_ = 0.269; F [2,106] = 20.76, *p* < 0.001).

The variation in knowledge had a stronger effect; for one more point in the knowledge delta, the safety-related behaviour score decreased by 0.017 points (b_1_ = −0.017, t = −5.87, *p* < 0.001, 95% CI −0.022–−0.011); for one more point in the safety climate–management action scale, the safety-related behaviour score decreased by −0.092 points (b_1_ = −0.092, t = −2.29, *p* = 0.024, 95% CI −0.017–−0.012). 

In Model 1, the knowledge delta alone was still fully significant and explained 24.7% of the variance of the procedures (R^2^ = 0.247, R^2^_adj_ = 0.241; F[2,103] = 34.9, *p* < 0.001; b1 = −0.017, 95% CI −0.023–−0.011).

## 4. Discussion

This study, which was based on a pre–post design, aimed to understand, in a sample of Italian mechanical workers, the effects of organizational factors (the management’s safety climate and colleagues’ safety climate), as well as individual factors (safety knowledge and attitudes), on safety-related behaviours. Research on safety in Italian mechanical companies is necessary due to the currently high rate of accidents. For this reason, to improve safety performance, we paid attention to the precursors of safe behaviours [4]. The results disconfirmed H3 and confirmed H1 and H2, showing that knowledge promotes not only the individually observed behaviours, but also does so, and more so, in combination with the management’s safety climate.

According to Griffin and Neal [6], the knowledge of the rules, regulations, procedures, and roles of the prevention system and unsafe behaviours defines what each worker must know to be able to act safely. These elements are critical to safety performance. Workers’ perception of the occupational risks appeared to be positively related to their comprehension of how important knowledge of rules and procedures was and, consequently, how much effort they put into becoming familiar with the relevant documentation. This stresses the need for ongoing safety education.

It is also important to consider that knowledge is more than information, as it involves a person’s awareness or understanding gained through experience, familiarity, or simulations. In this sense, it is necessary to reflect on the use of experiential methods producing situated learning. It would be easier, according to the action regulation theory [34], to learn safety rules by performing actions. This seems to be verified by our data, which were collected before and after the training, which was based on active teaching methods with the aim of facilitating learning through action. This intervention could help to link cognitive processes, behaviour, the external environment, and measurable results (e.g., safety performance) more effectively. However, no training intervention directly modifies behaviour, though it can eventually create the conditions for the expected behaviour to be implemented.

To understand the role of the social and organizational contexts in safety, researchers have identified the concept of the safety climate. Thus, this research also tested the role of the safety climate in activating safe behaviours. The data showed that the management’s safety climate contributed to the workers’ safe behaviour. On the other hand, in this study, the intra-group level linked to colleagues did not affect safety compliance (e.g., with the procedures and the correct use of PPE). The safety climate provided workers with information on the priority given by the organization to safety, translating it into standards of occupational behaviour. Our study demonstrated that the workers’ perception that safety was indeed a priority of the company management—which added to safety knowledge—increased the frequency of safe behaviours at the individual level. Therefore, the management’s safety climate increased the positive effect of knowledge on the production of safety compliance.

We believe that the colleagues’ safety climate needs further investigation. The lack of its effect on safety compliance could be connected with the sample, which was composed of workers who belonged to different working teams. Obviously, even within the same company, this result can depend on different safety perceptions, which are determined by different working conditions and team processes [30]. It would, therefore, be useful to investigate this specific effect at the level of each working team because workers are more likely to pay attention to colleagues’ safety habits and actions, such as real team rules, which, in turn, are affected by working conditions, as well as to their own team leader, who is seen as an effective safety leader [50].

The example that a team leader sets may or may not send the message to employees that workplace safety is a shared priority, which would motivate them to find ways to improve safety performance and support them in achieving that goal [9]. Classroom training alone may convey the message that the management values safety, but the same may not apply at the teamwork level through the day-to-day practices shared among colleagues. It should also be considered that, with each edition of the training in the companies involved in the study, workers from other organizational units that did not belong to the same work unit participated as well. Therefore, there was a lack of elements that would trigger a virtuous circle of mutual observation through which workers perceive the importance of their behaviour, even in influencing others [30]. We think that future research will likely consider colleagues’ safety climate as a precursor of safety participation [42], not of compliance. In fact, participation involves collaboration with colleagues to encourage safe work behaviours, showing initiative, engagement, and proactivity [8], which can help to promote a framework that considers safety as a primary value [9].

Our data showed that safety attitude was not predictive of safe behaviours. The workers’ “subjective” judgement of occupational risk probably had very broad inter-individual variability, which also depended on how the workers experienced traumatic events or accidents and the severity of the accidents [25]. Furthermore, some studies have indicated that safety attitude has a direct effect on safety participation; this is a more proactive behaviour, and it indirectly affects safety compliance, which is more related to knowledge of the procedures and rigorous application of the rules [24].

This ecological research has several limitations indeed: It involved a non-representative convenience sample, and the choice of measures depended on the agreement with the companies involved, with the cultural level of the participants, and with their familiarity with social research. Nevertheless, it might be useful to expand and improve the measures adopted to further analyse the effects of training on the different outcomes. However, it should be noted that more complete measures, which would have been more expensive because of the participants’ effort and time required, would not have been suitable. In quality research designs featuring studies on safety training’s effectiveness, including a meta-analytical data collection [16] of ninety-five quasi-experimental studies and a follow-up systematic review [15], it was shown that, as far as the methodological quality was concerned, there were too few good or fair studies assessing the effect of training on workers’ knowledge and attitudes. In general, this review found a general lack of high-quality randomized controlled trials in the area of safety training efficacy, with a modest number of studies (no more than six per outcome) of fair or good methodological quality. It is, therefore, not surprising that the subsequent meta-analysis [17] identified, out of 28 studies, only seven randomized controlled trials, five studies adopting a random sampling, and three non-randomized investigations comparing a training group and a control group. Again, the methodological quality was lower for studies assessing the effect of training on knowledge and attitudes. These limitations are not to be attributed to the superficiality of the researchers and practitioners, but they must be considered as opportunities to carry out complex investigations in the field wherever, however, and whenever possible.

Finally, future research should test the effect of colleagues’ safety climate on safety compliance in a sample that has, for some time, regularly performed structured activities, and their daily working behaviours should be checked.

## 5. Conclusions

Considering the necessary caution when generalizing results, it is important to highlight that this sampling used a census, that is, all of the workers of the chosen departments participated in the study in each step of the way. This is an important aspect, considering that, in this area of research, a very limited number of good or fair studies emerge in terms of methodological quality. In particular, the greatest methodological limits can be seen in the surveys relating to the effect of training on knowledge and attitudes. Even more so, it is necessary to note that, in our study, the measures of knowledge and behaviour were not self-perceived. Specifically, safety knowledge was measured through a test–retest related to the content covered in the training sessions. Additionally, safe behaviours were measured through observation of sequences of actions that were actually implemented by workers during everyday activities. In this sense, the items adopted before and after the training allowed for an outside objective observer to examine the workers’ real knowledge and behaviours connected with safety compliance in the job. We believe that this is an important strength, as it is very unusual for researchers not to adopt self-perceived measures, which can be affected by strong response biases.

Furthermore, the results of our study have important implications.

Firstly, with regards to active teaching methods, these interventions could help to effectively link cognitive processes, behaviour, external environment, and measurable results, facilitating learning by action. Therefore, tailored training consistent with the specific safety procedures of the organizations increases knowledge related to the expected safe behaviours.

Secondly, this research emphasised the importance of continuous training. Continuous learning helps workers to develop a constant practice of being aware of their roles and responsibilities to maintain safety at work. Frequent training allows workers to cultivate safety-conscious habits and incorporate these updates into their operations. Indeed, this study evidenced that updating safety knowledge increases safety compliance and, subsequently, improves safety performance.

Thirdly, this study showed the prominent role of supervisors and top managers in giving further relevance through their actions to the need to respect the company’s rules and procedures. Increased managerial safety commitment, such as concerns about workers’ safety participation activities and participation safety activities, will directly motivate workers to contribute to safety. In fact, the management’s commitment to safety and the effectiveness with which the top management prioritizes safety in an organization play a crucial role in workers’ involvement and influence the latter’s degree of understanding of the safety rules and procedures, the availability of safety information, and the directions regarding personal protective equipment. Thus, companies’ management has to make their safety-related objectives explicit and create the conditions to make such objectives attainable, thus adding to the knowledge of the contents so as to demonstrate that safety represents a core organizational value.

Fourthly, our study has made a useful and original contribution to a better understanding of whether and how safety knowledge is a precursor to safety performance by analysing safety compliance separately from safety participation. The impact of safety knowledge on safety compliance has been little examined by literature. Consequently, this study allows one to understand that the level of safety knowledge is significantly related to safety compliance and that there are positive relationships among safety training, safe behaviours, and compliance with safety procedures.

Certainly, several questions remain open and, on the basis of the results presented, future research should be directed toward understand how colleagues’ safety climate affects safety performance. Some evidence of this study shows that safety knowledge (at the individual level) and the management’s safety climate (at the organizational level) produce safety compliance. However, since safe behaviour is not a simple adherence to rules and procedures, we need to understand what aspects are involved in safety participation. In this sense, colleagues’ safety climate could represent the missing link at the group level.

## Figures and Tables

**Table 1:** Model selection referring to the “safety-related behaviours” as an outcome. The models emerging as the best in the model confidence set are in bold.

Model: Predictor(s)	df	Loglikelihood	AICc	AICc Delta	Weight
**M12: knowledge plus management safety climate**	**4**	−63.289	135.0	0.00	0.524
**M1: knowledge**	**3**	−63.270	137.1	2.16	0.178
M13: knowledge interacting with management safety climate	5	−65.928	138.1	3.12	0.110
M10: knowledge plus colleague safety climate	4	−65.327	139.0	4.08	0.068
M11: knowledge interacting with colleague safety climate	5	−65.317	141.2	6.26	0.023
M14: knowledge plus attitudes plus colleague safety climate plus management safety climate	8	−62.645	142.7	7.78	0.011
M8: knowledge plus attitudes	6	−65.007	142.8	7.88	0.010
M9: knowledge interacting with attitudes	17	−59.802	160.4	25.44	0.000
M7: management safety climate	3	−78.645	163.5	28.56	0.000
M6: colleague safety climate	3	−80.211	166.7	31.69	0.000
Null model	2	−81.278	166.7	31.70	0.000
M2: attitude, item 1	3	−80.671	167.6	32.61	0.000
M4: attitude, item 3	3	−80.919	168.1	33.10	0.000
M3: attitude, item 2	3	−81.192	168.6	33.65	0.000
M5: attitudes: item 1 + item 2 + item 3	5	−80.064	170.7	35.75	0.000

## Data Availability

The data presented in this study are available on request from the corresponding author. The data are not publicly available due to restrictions for privacy.
